# Expression of ACTR3 in cervical cancer and impact on immune cell infiltration and prognosis: A comprehensive analysis based on bulk RNA-Seq and single-cell RNA-Seq

**DOI:** 10.1097/MD.0000000000046316

**Published:** 2026-05-12

**Authors:** Hongdong Wang, Jing Zhang, Jianwei Li, Bohao Sun, Yichen Wu, Hao Wang, Xu Chen, Xiujuan Shang

**Affiliations:** a Lianyungang Maternal and Child Health Hospital, Lianyungang, Jiangsu, China; b Department of Pathology, Second Affiliated Hospital, School of Medicine, Zhejiang University, Hangzhou, Zhejiang, China; c Department of Clinical Laboratory, Children’s Hospital, Zhejiang University School of Medicine, National Clinical Research Center for Child Health, Hangzhou, China; d Department of Laboratory Medicine, Lianyungang Affiliated Hospital of Nanjing University of Chinese Medicine, Lianyungang, Jiangsu, China.

**Keywords:** ACTR3, cervical cancer, immune cell infiltration, PI3K/Akt/mTOR pathway, poor-prognosis predictor

## Abstract

**Background:**

PI3K/Akt/mTOR pathway is crucial in some cancers, but its relation with tumor-infiltrating immune cells in cervical squamous cell carcinoma and endocervical adenocarcinoma (CESC) is unanalyzed. This study aimed to determine if ACTR3 overexpression affects CC survival and explore its impact on the tumor immune microenvironment.

**Methods:**

Research investigated ACTR3 expression levels related to PI3K/Akt/mTOR pathway and its influence on tumor immunology and clinical outcomes. Methods included RNA-seq data analysis, immune cell infiltration evaluation, survival analysis, gene enrichment analysis, and single-cell RNA-seq data integration. ACTR3 expression in cervical cancer specimens was evaluated by immunohistochemistry.

**Results:**

Least absolute shrinkage and selection operator (LASSO) Cox regression identified ten key genes including ACTR3 with prognostic value. High ACTR3 expression correlated with poor outcomes, suggesting it as a prognostic biomarker. The prognostic model was validated by time-dependent receiver operating characteristic (ROC) curves for 1-, 3-, and 5-year survival. A nomogram combining ACTR3 expression and clinical parameters estimated patient survival. Single-cell RNA sequencing showed ACTR3-expressing immune cells in CESC include dendritic cells (DCs), T cells, and tissue stem cells.

**Conclusion:**

This research elucidated a distinct signature linked to the PI3K/Akt/mTOR signaling pathway, particularly focusing on ACTR3, which plays a role in both the onset and advancement of CESC. Moreover, ACTR3 has the potential to act as a prognostic biomarker for patients diagnosed with CESC, thereby offering novel perspectives for the development of clinical therapeutic approaches.

## 1. Introduction

Cervical squamous cell carcinoma (CESC) represents a significant health challenge, being the third most prevalent cancer among women globally, contributing to substantial morbidity and mortality rates.^[1,2]^ The disease imposes a considerable psychological and economic burden on patients and healthcare systems alike. Despite the availability of vaccines and screening methods, current diagnostic and therapeutic strategies are hindered by limitations such as inadequate early detection and suboptimal treatment outcomes. Consequently, there is a pressing need for innovative approaches to enhance patient prognosis, particularly through the identification of novel biomarkers. Previous studies have underscored the critical role of the PI3K/Akt/mTOR signaling pathway in various malignancies, including CESC, where its aberrant activation is implicated in tumorigenesis and progression.^[3–5]^ However, existing research has primarily focused on broad pathway analyses, often overlooking the specific gene-level interactions that may provide deeper insights into tumor behavior and patient outcomes.^[6–8]^ This gap highlights the necessity of our study, which aims to explore the expression of genes associated with the PI3K/Akt/mTOR pathway and their prognostic implications in CESC. By employing advanced bioinformatics techniques, we seek to identify key prognostic markers that could inform clinical decision-making and ultimately improve patient management.

The present study focuses on the construction of a prognostic risk model that identifies a distinct set of genes associated with the PI3K/Akt/mTOR signaling pathway, specifically in the context of CESC. Prior research has established the critical role of this pathway in various malignancies, highlighting its potential as a therapeutic target.^[9–11]^ The PI3K/Akt/mTOR signaling pathway plays an important role in the occurrence, development, and treatment of cervical cancer. Studies have shown that this pathway is significantly activated in cervical cancer tissues, with its protein and gene expression levels being higher than those in normal or precancerous tissues.^[12,13]^ For example, the levels of PI3K/Akt/mTOR proteins in the cancer tissues of cervical cancer patients are higher than those in healthy individuals and patients with cervical intraepithelial neoplasia, and the expression is more significant with increasing malignancy.^[12]^ The activation of this pathway can promote tumor progression through various mechanisms: promoting cell proliferation and survival: VEGFa enhances the growth and invasive ability of cervical cancer cells by activating the PI3K/Akt/mTOR pathway, while inhibiting this pathway can significantly reduce tumor activity.^[14]^ The growth factor PGRN increases cellular protein synthesis and transformation by activating the mTOR signaling pathway, while rapamycin (an mTOR inhibitor) can block this process and inhibit tumor formation both in vivo and in vitro.^[15]^ Regulation of metastasis and invasion: Activation of downstream effector molecules of the PI3K/Akt/mTOR pathway (such as MMP2/MMP3) can promote cancer cell migration and invasion.^[14]^ In addition, the inhibition of the SRSF3 gene significantly reduces the viability and metastatic ability of cervical cancer cells by blocking this pathway.^[16]^ Metabolic reprogramming: AKT inhibitors reduce glucose uptake and glycolysis in cervical cancer cells by inhibiting the PI3K/Akt/mTOR pathway, thereby decreasing cell survival rate.^[17]^ Clinical studies have shown that abnormal activation of this pathway is associated with the prognosis of cervical cancer. For example, high expression of the ADRA2A receptor is associated with better prognosis in patients by inhibiting the PI3K/Akt/mTOR pathway,^[18]^ while the ERBB3 gene regulates epithelial-mesenchymal transition (EMT) through this pathway and affects immune cell infiltration in the tumor microenvironment, thereby influencing patient survival.^[19]^ In addition, mutations in the PIK3CA gene (such as E545K, E542K) and inactivating mutations in PTEN (such as R233) are common in cervical cancer and may affect treatment response.^[17]^ Targeted therapies against this pathway have shown potential. For example, the PI3K inhibitor LY294002 can induce apoptosis in cervical cancer cells and shows a dose-dependent inhibitory effect in in vitro experiments^[13]^; phytochemicals (such as curcumin) enhance the sensitivity to chemotherapy and radiotherapy by inhibiting this pathway^[20]^; the combined use of AKT inhibitors (such as SC-66) with glycolysis inhibitors 2-DG can synergistically enhance antitumor effects.^[17]^ In summary, the PI3K/Akt/mTOR pathway is a key regulatory pathway in cervical cancer, and its potential as a prognostic biomarker and therapeutic target has been widely validated.^[21]^

ACTR3 is a gene closely related to the occurrence and development of cancer, showing important biological roles in various tumor types. According to literature reports, ACTR3 has been identified as a core therapeutic target in non-small cell lung cancer (NSCLC), with its high expression significantly associated with poor prognosis in patients.^[22]^ Research shows that ACTR3 may promote the conversion of CD4 + T cells into immunosuppressive iTr35 cells by regulating the IL-35 signaling pathway, thereby inhibiting NK cell activity and enhancing tumor immune evasion. In addition, the high expression of ACTR3 is also associated with the maintenance of the immunosuppressive state in the tumor microenvironment, which may be achieved by reducing the levels of interferon-γ and inhibiting the function of effector T cells. In pancreatic cancer, ACTR3 is listed as one of the risk genes associated with liquid-liquid phase separation, and its expression level can be used to construct prognostic models.^[23]^ Research through multi-omics analysis has found that high expression of ACTR3 is associated with high-risk subtypes in pancreatic cancer patients, who exhibit a stronger tendency for immune evasion and lower sensitivity to immunotherapy. Further analysis shows that ACTR3 may promote tumor progression by regulating cell cycle-related pathways (such as CCNA2) and extracellular matrix receptors (such as ITGB1). These findings suggest that ACTR3 could serve as a prognostic marker and therapeutic target across different cancer types, with mechanisms involving immune microenvironment regulation and abnormal activation of cell signaling pathways. However, the role of ACTR3 in cervical cancer remains unclear.

Our investigation reveals a significant elevation of ACTR3 expression in CESC tissues compared to normal counterparts, alongside a notable correlation with other PI3K/AKT/mTOR related genes (PAMRGs). In this study, we employed a multifaceted approach integrating bioinformatics analysis, least absolute shrinkage and selection operator (LASSO) regression, and Cox regression analysis to construct a prognostic risk model focused on CESC. The advantage of this methodology lies in its ability to leverage large-scale genomic data to identify key prognostic genes associated with the PI3K/Akt/mTOR signaling pathway, thereby facilitating the development of a predictive model for patient outcomes. The primary objective of this research is to establish a risk-scoring system based on the expression levels of critical genes, including ACTR3, which can effectively predict overall survival rates in patients diagnosed with CESC. By elucidating the role of these genes in tumor biology, this study aims to enhance prognostic accuracy and inform clinical decision-making, ultimately contributing to improved patient management and therapeutic strategies in cervical cancer.

## 2. Materials and methods

### 2.1. Gene expression profiles

RNA sequencing datasets of normal and tumor tissue samples were obtained from The Cancer Genome Atlas (TCGA) (http://cancergenome.nih.gov) and the genotype-tissue expression (GTEx) (http://commonfund.nih.gov/GTEx/) project. A thorough compilation of 105 genes linked to the PI3K/AKT/mTOR signaling pathway, collectively designated as PAMRGs, was meticulously curated from the ‘HALLMARK_PI3K_AKT_MTOR_SIGNALING’ gene set available in the Molecular Signatures Database (MSigDB) (https://www.gsea-msigdb.org/gsea/msigdb), as detailed in Table S1 (Supplemental Digital Content, Supplemental Digital Content, https://links.lww.com/MD/Q827). Additionally, the single-cell RNA sequencing dataset designated as GSE168652 was obtained from the GEO database.

### 2.2. Development of a risk model associated with the PI3K/Akt/mTOR signaling pathway

The genes associated with prognosis were subjected to additional analysis through the least absolute shrinkage and selection operator (LASSO) method, utilizing the “glmnet” package.^[24]^ The patient’s characteristic gender and age are included as covariates in the regression model. This approach was implemented to minimize redundancy and mitigate the risk of overfitting the model. Subsequently, a set of ten genes was selected to construct a prognostic risk-scoring model aimed at forecasting overall survival (OS) in patients diagnosed with CESC.

### 2.3. Survival analysis

We undertook a series of assessments to confirm the accuracy of the proportional hazards assumption in relation to Cox regression analysis, employing the “survival” package for this purpose. Subsequently, we created visual representations in the format of forest plots utilizing the “ggplot2” package. The Kaplan–Meier (KM) analysis was executed through the “survival” package to both assess the proportional hazards assumption and to develop survival regression models.^[24]^ Furthermore, we explored the prognostic significance of ACTR3 by examining clinical data obtained from the TCGA database.

### 2.4. Analysis of differentially expressed mRNAs

In this study, the dataset was subjected to normalization and log transformation, specifically using transcripts per million (TPM) methodology. The normalization process is specifically characterized by the exclusion of genes exhibiting significantly low expression levels, based on their expression profiles across the samples. In addressing missing values, we opt to eliminate genes that present such deficiencies. For each gene under consideration, we compute the mean and standard deviation across all samples. The final step involves the application of a log_2_ transformation to mitigate the skewness inherent in the dataset. Following this, the “limma” package was utilized, implementing the criteria of |logFC| > 1 and *P*.adj < .05.^[25]^ To facilitate visualization, a volcano plot was created to depict the mRNA data, in conjunction with a heatmap that displayed the target gene alongside its co-expressed mRNAs, employing the “ggplot2” package for the graphical illustrations.

### 2.5. Gene enrichment analysis

The samples were divided into 2 groups, namely high-expression and low-expression cohorts, based on the median expression level of the ACTR3 gene. To analyze the differentially expressed genes, enrichment analyses were conducted utilizing gene ontology (GO) and the Kyoto Encyclopedia of Genes and Genomes (KEGG) through the R package known as “clusterProfiler.”^[26]^ Additionally, gene set enrichment analysis (GSEA) was performed using the same “clusterProfiler” package. Notable results were determined by identifying gene sets that exhibited a normalized enrichment score (NES) >1, alongside a false discovery rate (FDR) of <0.05.

### 2.6. Development of nomograms

Nomograms that integrate the risk score with the ACTR3 models were created employing the “rms” R package to forecast OS in CESC by utilizing data sourced from the TCGA cohort.^[27]^ To evaluate the predictive accuracy of these nomograms, a thorough assessment was performed through the use of time-dependent calibration curves, enabling a comparison between the predicted outcomes and the actual survival data across a designated time interval.

### 2.7. Immune infiltration analysis

The data failed to satisfy the normality assumption; consequently, spearman correlation analysis was performed to examine the association between the expression of ACTR3 and the abundance of various infiltrating immune cells present in the tumor microenvironment, with a significance level established at *P* < .05. To assess the levels of immune infiltration, we applied the ssGSEA algorithm from the R package “GSVA,” leveraging immune cell markers recognized in pertinent immunological research.^[28]^

### 2.8. Single-cell RNA-seq data integration and analysis

The dataset of single-cell RNA sequencing designated as GSE168652 was processed utilizing the “Seurat” package within the R programming framework. A comprehensive set of quality control protocols was executed to filter the cells, which included a criterion of maintaining a mitochondrial UMI ratio below 10%. These protocols were crucial for the exclusion of inferior cells, thereby facilitating the establishment of a trustworthy and extensive dataset for future analyses. The normalized data were subsequently combined utilizing the LogNormalization approach. Subsequently, cell clustering analysis was performed using the “FindNeighbors” and “FindClusters” functions.^[29,30]^ To illustrate the resulting cell clusters, techniques such as PCA, t-SNE, and UMAP were employed. The annotations pertaining to cell types were obtained by extracting cell cluster markers from the CellMarker 2.0 database (http://bio-bigdata.hrbmu.edu.cn/CellMarker/).^[31,32]^

### 2.9. Patients and tissue specimens

An immunohistochemical (IHC) assessment was performed on samples collected from 45 patients diagnosed with cervical cancer at the Second Affiliated Hospital of Zhejiang University School of Medicine, aiming to investigate the varied expression levels of ACTR3. This research received approval from the Ethics Committee of the Second Affiliated Hospital of Zhejiang University School of Medicine (approval reference number 2025-0261). Furthermore, the Ethics Committee granted authorization for the study while waiving the necessity for obtaining informed consent from the patients involved.

### 2.10. Immunohistochemistry analysis

Tissue sections, measuring 2.5 µm in thickness, were affixed to glass microscope slides and subjected to deparaffinization using xylene, followed by rehydration through a series of graded alcohols. The sections underwent antigen retrieval by being boiled for 15 minutes in a 10 mM citric acid buffer at pH 6.0. Once cooled to room temperature, the sections were treated with 3% hydrogen peroxide for 15 minutes at 37°C to inhibit any endogenous peroxidase activity. Subsequently, the sections were exposed to primary antibodies, specifically ACTR3 (dilution of 1:1000; Proteintech, Wuhan, China) and KI67, CK7, p40, p16, and p63 (dilution of 1:1000; Zhongshan Golden Bridge Biotechnology Co., Beijing, China), for an overnight incubation at 4°C. Following this, the sections were incubated with secondary antibodies, including goat anti-rabbit IgG (1:1000; Abcam, Cambridge), for a duration of 30 minutes. Finally, color development was achieved using a diaminobenzidine substrate kit (Abcam), and the sections were counterstained with hematoxylin for 30 seconds.

### 2.11. Statistical analysis

Statistical evaluations and bioinformatics analyses were conducted utilizing R software (version 4.4.0). In the evaluation of pathological TNM staging, an initial assessment of the data’s normality was performed. This was subsequently followed by the implementation of the analysis of variance (ANOVA) technique, after which a Tukey confidence interval analysis was carried out. The Wilcoxon rank-sum test was employed to compare the 2 groups, as the data exhibited a non-normal distribution. Additionally, in the correlation analysis, the Spearman rank correlation method was employed due to the data’s deviation from a normal distribution. For the purpose of analyzing survival data, the KM method was utilized.

## 3. Results

### 3.1. Construction of prognostic risk model

**Figure 1.**
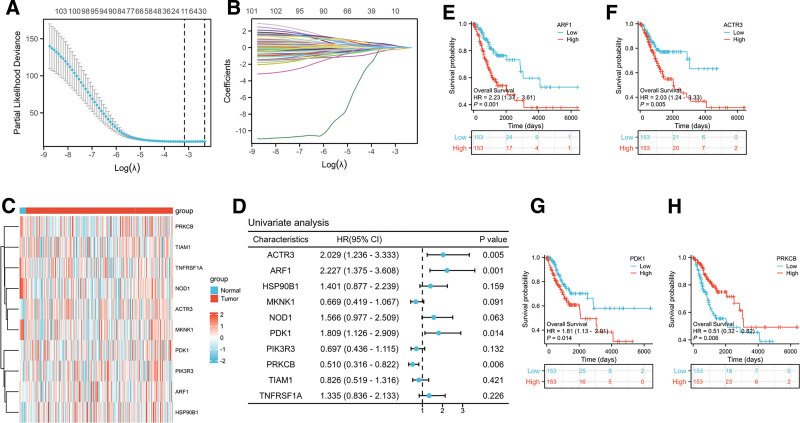
Construction and validation of the PI3K/Akt/mTOR-related risk model based on the TCGA cohort. (A) The LASSO regression analysis revealed ten hub genes that serve as significant factors in the risk assessment model. (B) The coefficient profiles obtained from the LASSO regression analysis for the 105 genes associated with the PI3K/Akt/mTOR signaling pathway. (C) The heatmap offers a graphical depiction of the expression profiles of PAMRGs. (D) The forest plot illustrates the prognostic significance of genes associated with the PI3K/Akt/mTOR signaling pathway. (E–H) Kaplan–Meier survival analysis is employed to assess the relationship between the expression levels of PAMRGs and patient outcomes. LASSO = least absolute shrinkage and selection operator, PAMRGs = PI3K/AKT/mTOR related genes, TCGA = The Cancer Genome Atlas.

We developed a prognostic risk model and identified a specific set of genes associated with the PI3K/Akt/mTOR signaling pathway for subsequent analysis. Utilizing LASSO Cox regression analysis, we successfully pinpointed ten crucial genes that hold prognostic significance: ACTR3, ARF1, HSP90B1, MKNK1, NOD1, PDK1, PIK3R3, PRKCB, TIAM1, and TNFRSF1A (Fig. 1A and B, Table S2, Supplemental Digital Content, https://links.lww.com/MD/Q828). To evaluate the expression profiles of these ten prognostic metabolic risk genes, a comparative analysis was conducted between CESC tissues and normal tissue samples. The results were further substantiated by the gene expression heatmap presented (Fig. 1C). A univariate Cox regression analysis was performed to ascertain the prognostic significance of the ten PAMRGs (Fig. 1D). Furthermore, we conducted a log-rank test to evaluate the statistical significance of the disparities observed between the Kaplan–Meier curves. Kaplan–Meier survival analysis revealed that patients exhibiting low expression levels of ARF1, ACTR3, and PDK1 had a notable survival advantage compared to those with higher expression levels of these genes (Fig. 1E–G). In contrast, PRKCB exhibited a contrasting trend (Fig. 1H).

### 3.2. The mRNA levels of expression and the interrelationship of PAMRGs in CESC

**Figure 2.**
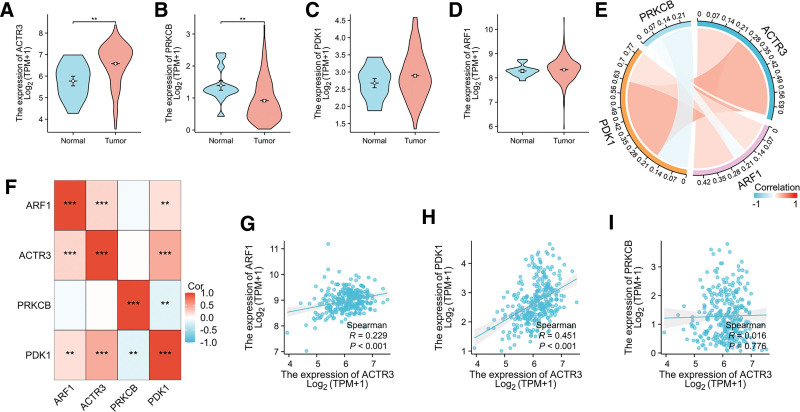
The expression and correlation of PAMRGs in tumor tissues. (A–D) Comparison of PAMRG expression levels between the normal group and the CESC group. (E) The chord diagram depicts the interconnections between PAMRGs. (F) The heatmap displays the correlations between the PAMRGs. (G–I) The scatter plot demonstrates the correlation between the PAMRGs. CESC = cervical squamous cell carcinoma and endocervical adenocarcinoma, PAMRGs = PI3K/AKT/mTOR related genes.

The expression levels of ACTR3 are significantly elevated in CESC, as depicted in Figure 2A. This observation sharply contrasts with the pronounced downregulation of PRKCB, which is also observed within the same context (Fig. 2B). In contrast, the expression levels of ARF1 and PDK1 show no noteworthy changes (Fig. 2C and D). Subsequently, we performed a correlation analysis to evaluate the interactions among these critical genes. Given that the data did not fulfill the criteria for normal distribution, we employed Spearman correlation analysis to discern the relationships among these genes (Fig. 2E and F). Importantly, a significant positive correlation was observed between ACTR3 and both ARF1 and PDK1 (Fig. 2G and H). Conversely, no correlation was detected between ACTR3 and PRKCB (Fig. 2I).

### 3.3. ACTR3 expression is significantly elevated in various cancers, including CESC

**Figure 3.**
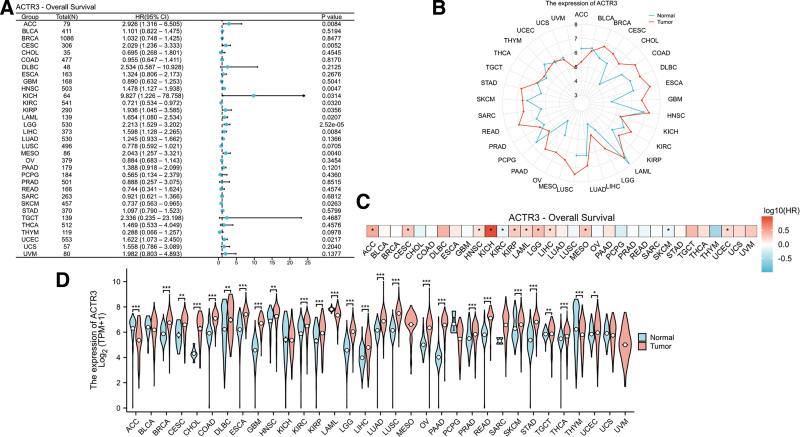
The expression of ACTR3 is notably elevated in various cancers, including CESC. (A) The relationship between the expression levels of ACTR3 and OS in patients with various cancer types was assessed through univariate Cox regression analysis. (B) The radar chart depicts the expression levels of ACTR3 in tumor tissues. (C) A heatmap analysis was conducted to evaluate the correlation between the expression levels of ACTR3 and OS in patients with various cancer types. (D) The expression levels of ACTR3 in various human cancer tissues were analyzed in comparison to those in normal tissues, utilizing data sourced from the TCGA and GTEx databases. GTEx = genotype-tissue expression, OS = overall survival, TCGA = The Cancer Genome Atlas.

In order to explore the possible differences and outcomes linked to ACTR3 expression between malignant tissues and their adjacent normal tissues, we performed an analysis utilizing data from 33 different cancer types obtained from TCGA and the GTEx project. A univariate Cox regression analysis was conducted to evaluate the relationship between ACTR3 expression levels and OS across these cancer types (Fig. 3A). The results revealed that elevated ACTR3 expression is significantly correlated with negative outcomes in patients diagnosed with adrenocortical carcinoma (ACC), CESC, head and neck squamous cell carcinoma (HNSC), chromophobe renal cell carcinoma (KICH), clear cell renal cell carcinoma (KIRC), papillary renal cell carcinoma (KIRP), acute myeloid leukemia (LAML), lower grade glioma (LGG), hepatocellular carcinoma (LIHC), mesothelioma (MESO), skin cutaneous melanoma (SKCM), and endometrial carcinoma (UCEC). These results are additionally supported by the heatmap illustration presented in Figure 3C. The evaluation of ACTR3 expression across various cancer datasets underscores its importance in tumor biology, with a notable upregulation detected in the majority of cancer types, particularly in CESC (*P* < .01, Fig. 3B and D).

### 3.4. Expression and prognosis of ACTR3-related proteins

**Figure 4.**
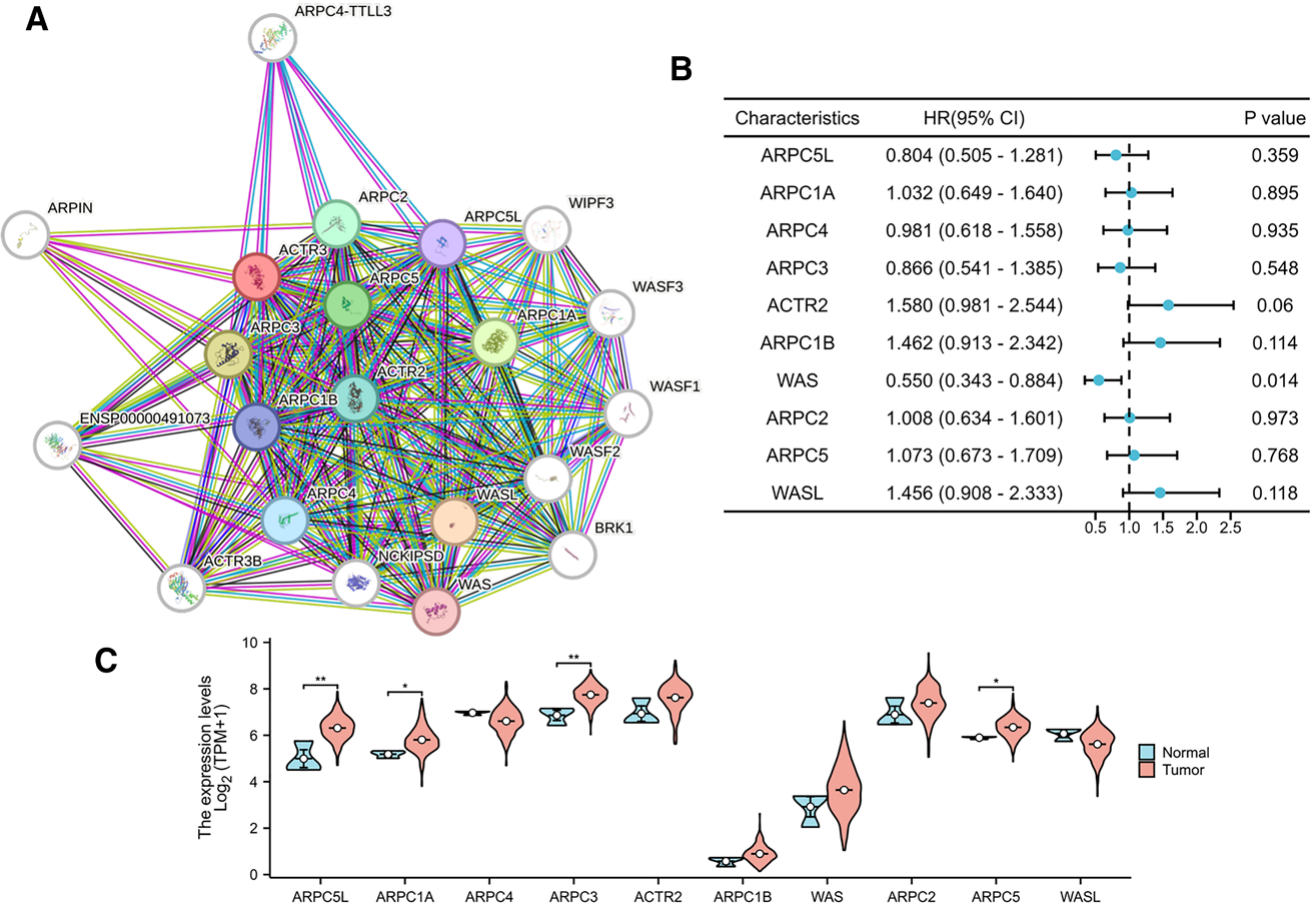
Expression of ACTR3-related genes and their prognostic significance. (A) A schematic representation illustrating the relationships among ACTR3 and several proteins obtained from the STRING database. (B) The forest plot illustrates the significance of genes associated with ACTR3 in terms of their predictive value. (C) The comparison of mRNA expression levels of ACTR3-related genes was conducted between normal tissues and tumor tissues.

**Figure 5.**
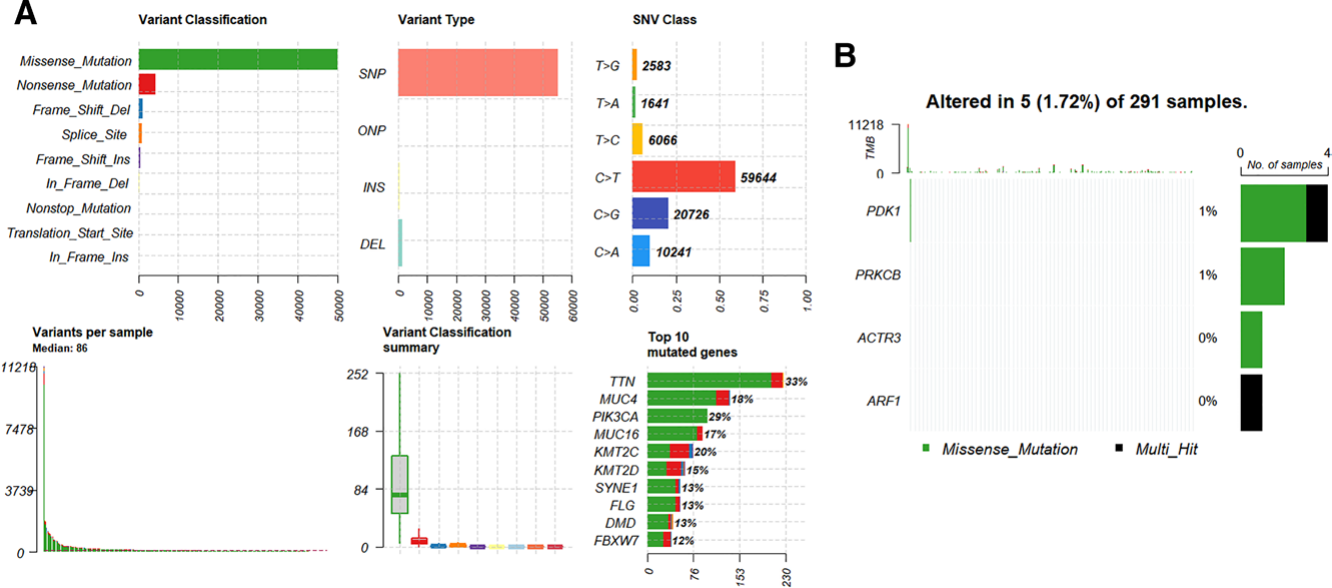
Gene mutation analysis. (A) The status of mutations associated with CESC as documented in the TCGA database. (B) Mutational profile of key molecules in 291 cervical cancer tissue samples. CESC = cervical squamous cell carcinoma and endocervical adenocarcinoma, TCGA = The Cancer Genome Atlas.

A protein–protein interaction (PPI) network was established using the STRING database to investigate the proteins interacting with ACTR3 (Fig. 4A). The ten genes identified (ARPC5L, ARPC1A, ARPC4, ARPC3, ACTR2, ARPC1B, WAS, ARPC2, ARPC5, and WASL) exhibited a significant association with the functional role of ACTR3. To evaluate the prognostic relevance of these genes, a univariate Cox regression analysis was conducted, with the findings summarized in Figure 4B. The analysis indicated that the expression levels of WAS are significantly correlated with patient outcomes in CESC. Additionally, it was observed that the expression levels of ARPC5L, ARPC1A, ARPC3, and ARPC5 were notably elevated in CESC cases (Fig. 4C and D). The waterfall plot depicted in Figure 5A reveals that missense mutations were the most frequently occurring type. However, mutations within the ACTR3 gene were not detected in cervical cancer cases (Fig. 5B).

### 3.5. Functional enrichment analysis of ACTR3 in CESC

**Figure 6.**
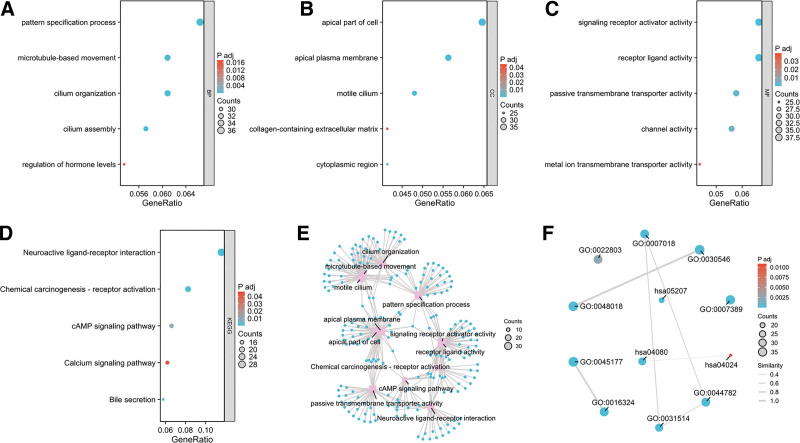
Analysis of functional enrichment concerning DEGs predicated on the expression levels of ACTR3. (A–C) The GO enrichment analysis conducted on DEGs associated with ACTR3 identified significant enrichment in various biological processes, cellular components, and molecular functions. (D) An analysis of KEGG pathway enrichment pertaining to ACTR3 in CESC was conducted. (E–F) The grid chart presents the results obtained from the GO and KEGG analyses. CESC = cervical squamous cell carcinoma and endocervical adenocarcinoma, DEGs = differential expressed genes, GO = gene ontology, KEGG = Kyoto Encyclopedia of Genes and Genomes.

The functional enrichment analysis utilizing GO identified noteworthy correlations of ACTR3 with a range of molecular functions (MF), cellular components (CC), and biological processes (BP) (Table 1). Specifically, it was determined that ACTR3 plays a role in various biological processes, such as the pattern specification process, ciliary organization, microtubule-based movement, ciliary assembly, and the modulation of hormone levels (Fig. 6A, E, and F). Regarding cellular components, ACTR3 was associated with structures including the apical region of the cell, the apical plasma membrane, motile cilia, the cytoplasmic area, and the extracellular matrix rich in collagen (Fig. 6B, E, and F). Furthermore, the analysis of molecular functions revealed that ACTR3 is involved in activities such as ligand-receptor interactions, activation of signaling receptors, passive membrane transport, channel activity, and the translocation of metal ions across membranes (Fig. 6C, E, and F). In addition, KEGG pathway analysis highlighted the co-enrichment of ACTR3 along with its co-expressed mRNAs in several pathways, including neuroactive ligand-receptor interactions, chemical carcinogenesis via receptor activation, as well as the cAMP and calcium signaling pathways, in addition to bile secretion (Fig. 6D, E, and F).

**Table 1:** Supplementary information on the results of GO and KEGG analyses.

Otology	ID	Description	*P* value
BP	GO:0007389	Pattern specification process	7.53415E−08
BP	GO:0044782	Cilium organization	2.52061E−08
BP	GO:0007018	Microtubule-based movement	4.96091E−08
BP	GO:0060271	Cilium assembly	4.66956E−08
BP	GO:0010817	Regulation of hormone levels	.000269937
CC	GO:0045177	Apical part of cell	1.2902E−09
CC	GO:0016324	Apical plasma membrane	6.74532E−09
CC	GO:0031514	Motile cilium	9.4683E−11
CC	GO:0099568	Cytoplasmic region	5.02256E−07
CC	GO:0062023	Collagen-containing extracellular matrix	.001865279
MF	GO:0048018	Receptor ligand activity	2.24163E−07
MF	GO:0030546	Signaling receptor activator activity	3.20699E−07
MF	GO:0022803	Passive transmembrane transporter activity	2.84374E−05
MF	GO:0015267	Channel activity	6.49275E−05
MF	GO:0046873	Metal ion transmembrane transporter activity	.001993588
KEGG	hsa04080	Neuroactive ligand-receptor interaction	2.75548E−06
KEGG	hsa05207	Chemical carcinogenesis – receptor activation	4.30287E−06
KEGG	hsa04024	Camp signaling pathway	.000851717
KEGG	hsa04020	Calcium signaling pathway	.005074782
KEGG	hsa04976	Bile secretion	2.9281E−07

BP = biological processes, CC = cellular components, GO = gene ontology, KEGG = Kyoto Encyclopedia of Genes and Genomes, MF = molecular functions.

**Figure 7.**
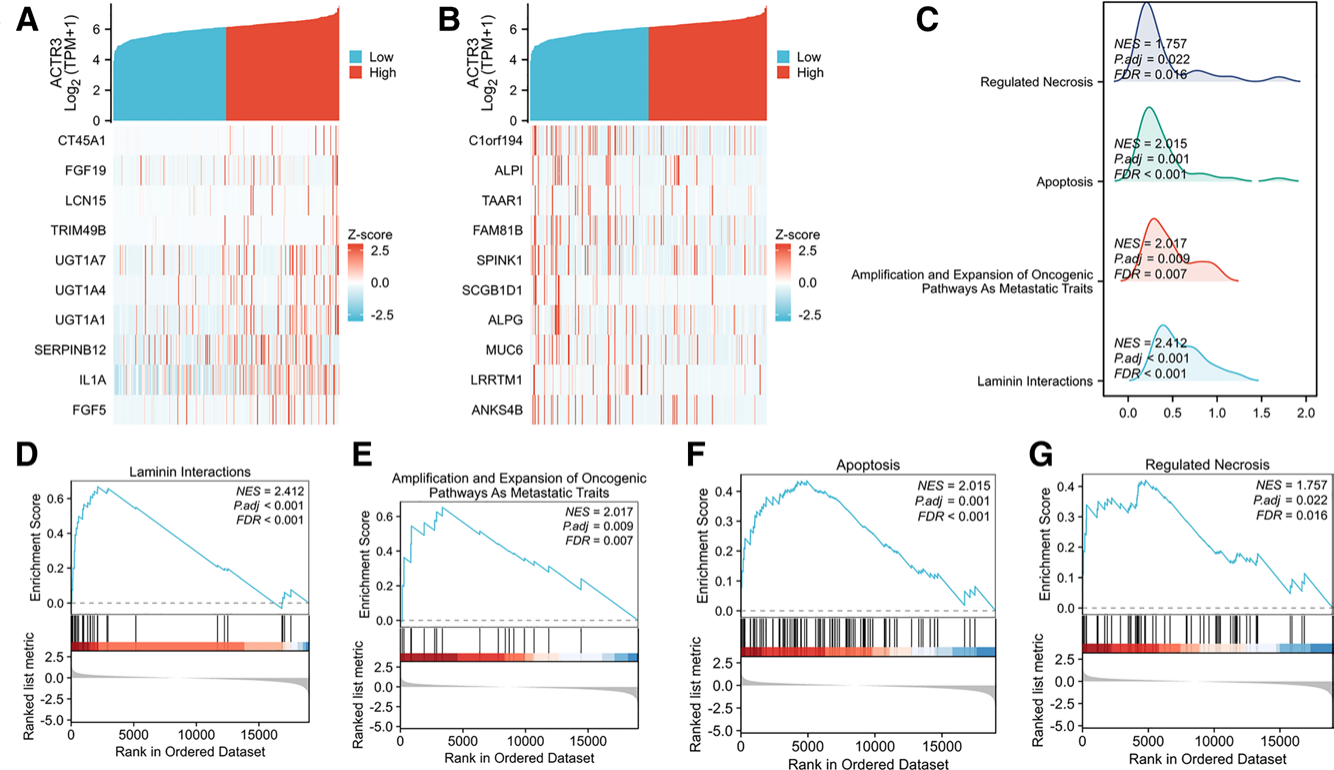
GSEA enrichment analysis of ACTR3. (A) The heat maps illustrate the ten genes that exhibit a positive co-expression relationship with ACTR3. (B) The heat maps illustrate the ten genes that exhibit a negative co-expression relationship with ACTR3. (C) The results obtained from GSEA are illustrated using mountain plots. (D–G) An examination of the functional roles and pathway enrichment related to ACTR3 is provided. GSEA = gene set enrichment analysis.

The heat maps depicted in Figure 7A and B showcase 10 genes that displayed both positive and negative correlations with ACTR3. In addition, we performed GSEA, which uncovered a wide array of biological processes linked to these genes. These processes included interactions with laminins, the upregulation and activation of oncogenic pathways typical of metastatic behavior, along with mechanisms associated with apoptosis and regulated necrosis (Fig. 7C–G).

### 3.6. Relationship between ACTR3 and immune infiltration

**Figure 8.**
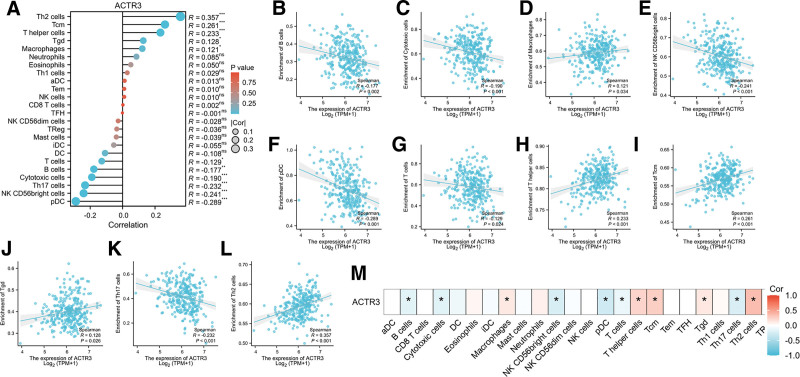
The examination of the relationship between the infiltration of immune cells and the expression levels of ACTR3. (A) Spearman correlation analysis was conducted to evaluate the relationship between the infiltration levels of 24 distinct immune cell types and the expression levels of ACTR3. (B–L) A scatter plot illustrating the extent of immune cell infiltration in relation to varying levels of ACTR3 expression. (M) A heatmap depicting the infiltration of immune cells corresponding to different levels of ACTR3 expression.

We investigated the correlations between the expression of ACTR3 and 24 distinct categories of immune cells, revealing a significant presence across the majority of immune cell infiltrates. The interactions that transpire between immune cells and tumor cells in the tumor microenvironment are pivotal to the progression of CESC. A thorough evaluation of the infiltration levels of these 24 immune cell types within CESC tissues was conducted utilizing ssGSEA. Furthermore, given that the data exhibited a non-normal distribution, we employed the Spearman correlation coefficient to quantitatively evaluate the association between ACTR3 expression levels and the infiltration of immune cells. The findings indicated a negative correlation between ACTR3 expression and 6 specific immune cell types, including T cells (*R* = −0.129), B cells (*R* = −0.177), cytotoxic cells (*R* = −0.190), Th17 cells (*R* = −0.232), NK CD56 bright cells (*R* = −0.241), and plasmacytoid dendritic cells (pDC) (*R* = −0.289) (Fig. 8A–M).

### 3.7. Clinical prognostic value of ACTR3 in CESC

**Figure 9.**
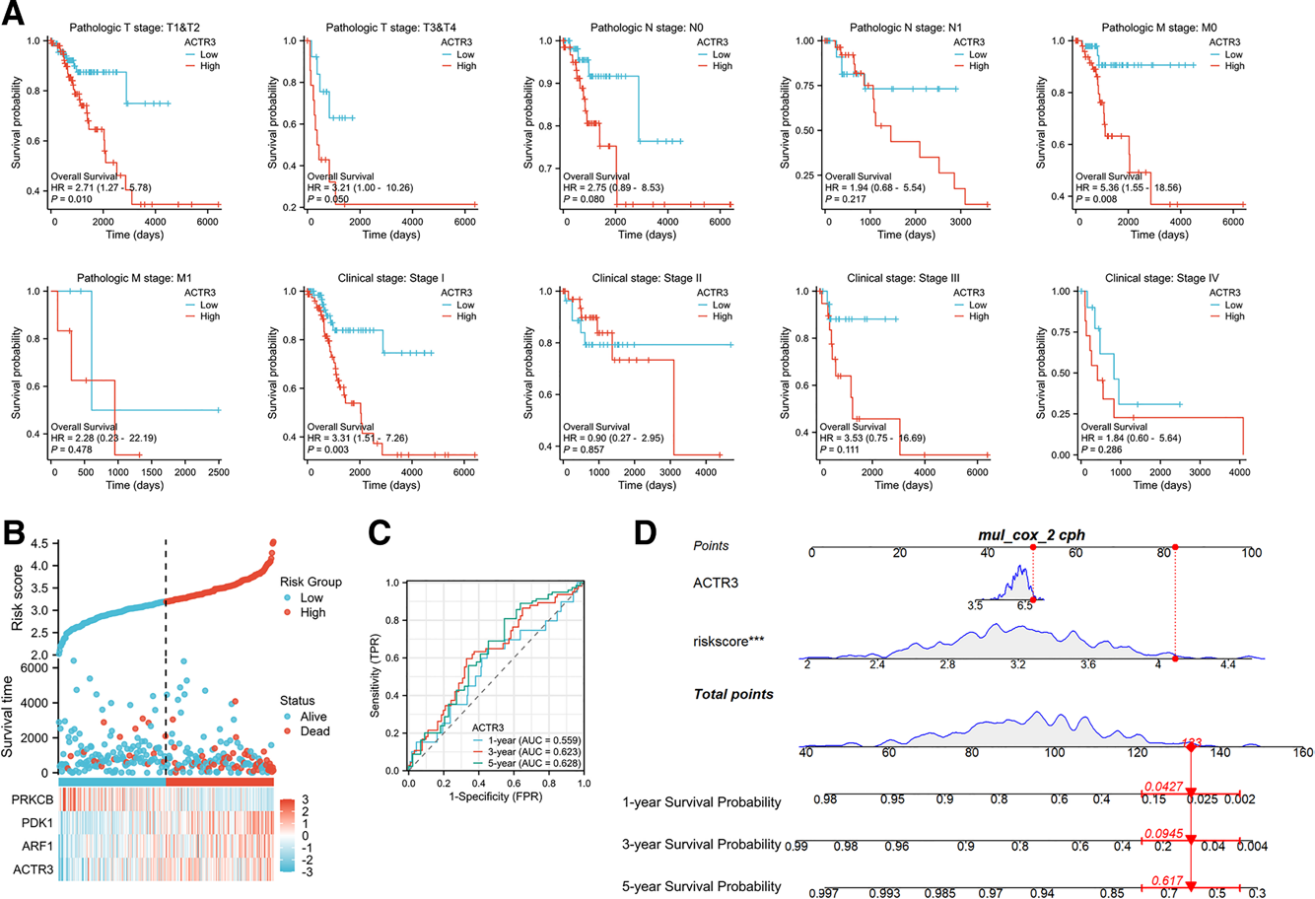
The influence of ACTR3 expression on prognosis and its diagnostic significance. (A) The relationship between OS and the expression levels of ACTR3 across different clinical subgroups of CESC. (B) Proportion of mortality as risk score values escalated within low and high-risk groups. (C) Time-dependent survival ROC curves were generated to estimate the survival probabilities at 1-, 3-, and 5-yr intervals for patients diagnosed with CESC, utilizing the expression levels of ACTR3 as the predictive variable. (D) A nomogram was constructed to forecast the OS rates at 1, 3, and 5 yr for individuals diagnosed with CESC. CESC = cervical squamous cell carcinoma and endocervical adenocarcinoma, OS = overall survival, ROC = receiver operating characteristic.

In the context of CESC research, the levels of ACTR3 expression have been identified as having a significant correlation with patient prognoses. Consequently, this investigation aims to further explore the association between ACTR3 expression and outcomes among different clinical subgroups. The findings derived from the Kaplan–Meier survival curve analysis, utilizing the log-rank test, indicate that heightened expression levels of ACTR3 are markedly correlated with a reduced overall survival across multiple clinical cohorts. This association is particularly evident in patients categorized as stage T1, T2, and M0, in addition to those identified with pathological stage I (Fig. 9A).

### 3.8. Association of ACTR3 expression with clinical parameters in CESC

In addition, we performed an analysis of the expression patterns of 4 PAMRGs genes in relation to risk scores, survival duration, and patient survival status using the TCGA dataset. Our results underscored the notable prognostic significance of ACTR3 (Fig. 9B). The time-dependent receiver operating characteristic (ROC) curves demonstrated area under the curve (AUC) values for the respective time points of 1, 3, and 5 years, further validating the robust performance of the model (Fig. 9C). Moreover, we constructed column-line plots that illustrate the OS prognostic outcomes, thereby elucidating the connection between ACTR3, risk score, and the probabilities of survival over 1, 3, and 5 years (Fig. 9D).

**Figure 10.**
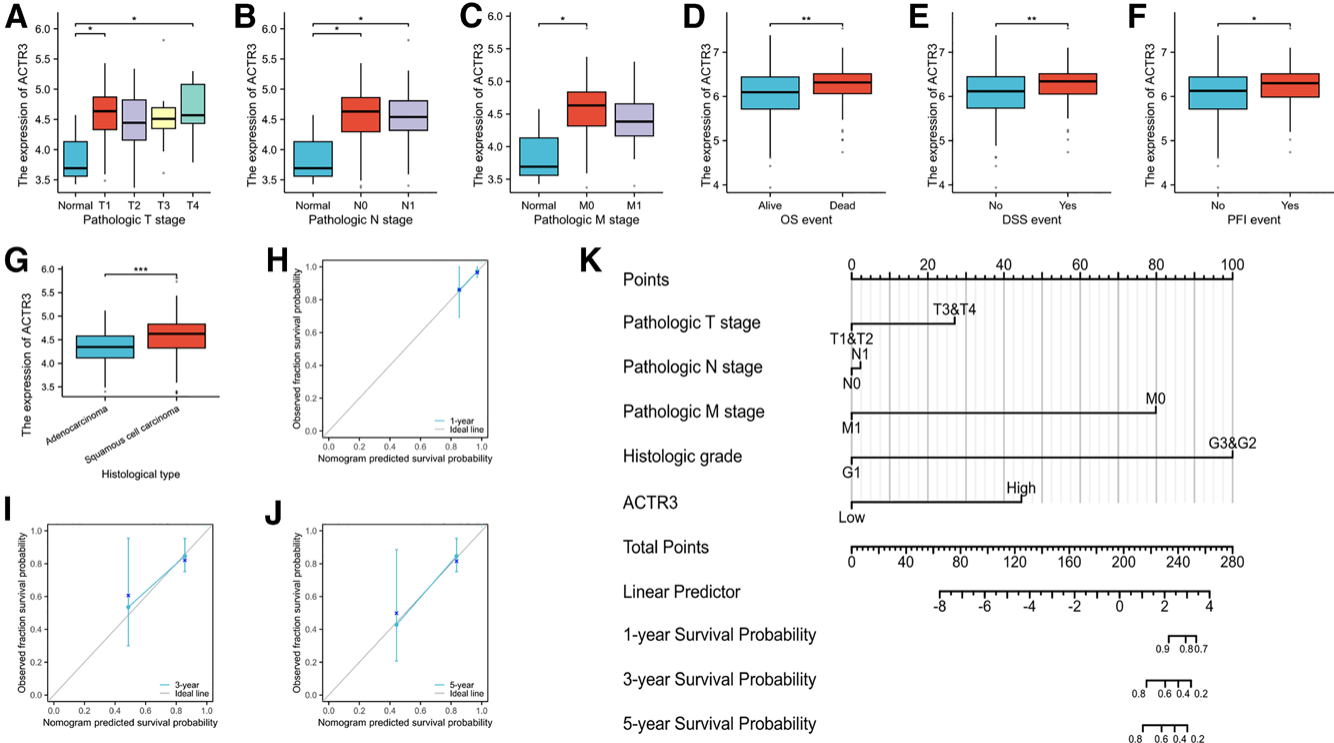
Association between ACTR3 expression and clinical features. (A–G) The correlation between ACTR3 expression and multiple clinical parameters – such as the T stage, N stage, M stage, OS events, DSS events, PFI events, and histological type – have been explored. (H–J) Calibration plots were generated to assess the accuracy of predictions for OS at 1-, 3-, and 5-yr intervals. (K) A nomogram has been developed to forecast the 1-, 3-, and 5-yr OS rates in patients diagnosed with CESC. CESC = cervical squamous cell carcinoma and endocervical adenocarcinoma, DSS = disease-specific survival, OS = overall survival, PFI = progression-free interval.

Our analysis revealed that the expression levels of ACTR3 exhibited a marked increase correlating with advanced T, N, and M stages (Fig. 10A–C). Furthermore, elevated ACTR3 expression was found to be significantly associated with OS event, disease-specific survival (DSS), and progression-free interval (PFI) events (Fig. 10D–F). Consequently, patients presenting with lower levels of ACTR3 expression demonstrated a notable survival advantage. Noteworthy is the observation that ACTR3 expression in cervical squamous carcinoma was significantly greater compared to that in cervical adenocarcinoma (Fig. 10G). Additionally, similar results were corroborated through the application of the χ^2^ test (Table 2). These results indicate a significant association between ACTR3 expression, clinical characteristics, and patient prognosis in CESC. Subsequently, we synthesized T stage, N stage, M stage, histological grade, and ACTR3 expression to develop a nomogram aimed at predicting survival outcomes (Fig. 10K). The calibration plot demonstrated that the nomogram’s predictive accuracy was reliable (Fig. 10H–J). Importantly, ACTR3 expression has the potential to improve the precision of survival probability predictions at the 1-year, 3-year, and 5-year marks. In summary, a significant relationship has been established between ACTR3 expression levels and prognostic outcomes in CESC.

**Table 2:** The clinicopathological characteristics identified in CESC patients with elevated versus reduced ACTR3 expression levels.

Characteristics	Low expression of ACTR3	High expression of ACTR3	*P* value
n	153	153	
Pathologic T stage, n (%)			
T1	60 (24.7%)	80 (32.9%)	.044
T2	41 (16.9%)	31 (12.8%)	
T3	15 (6.2%)	6 (2.5%)	
T4	5 (2.1%)	5 (2.1%)	
Pathologic N stage, n (%)			
N0	59 (30.3%)	75 (38.5%)	.192
N1	33 (16.9%)	28 (14.4%)	
Pathologic M stage, n (%)			
M0	53 (41.7%)	63 (49.6%)	.086
M1	8 (6.3%)	3 (2.4%)	
Clinical stage, n (%)			
Stage I	70 (23.4%)	92 (30.8%)	.015
Stage II	32 (10.7%)	37 (12.4%)	
Stage III	31 (10.4%)	15 (5%)	
Stage IV	14 (4.7%)	8 (2.7%)	
Age, n (%)			
≤50	91 (29.7%)	97 (31.7%)	.481
>50	62 (20.3%)	56 (18.3%)	
OS event, n (%)			
Alive	129 (42.2%)	105 (34.3%)	.001
Dead	24 (7.8%)	48 (15.7%)	
DSS event, n (%)			
No	133 (44%)	114 (37.7%)	.010
Yes	19 (6.3%)	36 (11.9%)	
PFI event, n (%)			
No	124 (40.5%)	110 (35.9%)	.059
Yes	29 (9.5%)	43 (14.1%)	
Histologic grade, n (%)			
G1	13 (4.8%)	6 (2.2%)	.230
G2	65 (23.8%)	70 (25.6%)	
G3	57 (20.9%)	62 (22.7%)	

CESC = cervical squamous cell carcinoma and endocervical adenocarcinoma, DSS = disease-specific survival, OS = overall survival.

### 3.9. Single-cell RNA sequencing analysis and clustering

**Figure 11.**
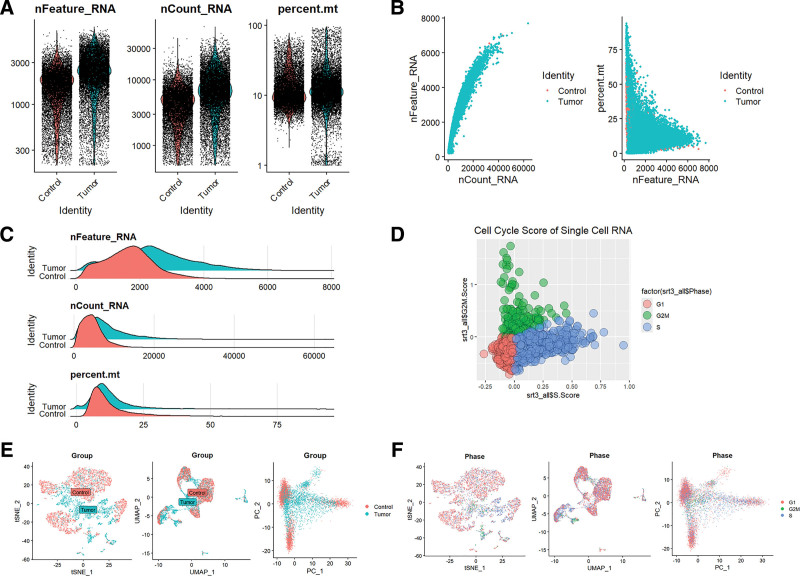
Mitigating the influence of mitochondrial DNA and the cell cycle on the analysis of single-cell data. (A–C) The evaluation of RNA features includes nFeature_RNA, nCount_RNA, and the percentage of mitochondrial content. (D) Assessment of cell cycle scores. (E) An examination of cellular aggregation throughout different phases of the group and cell cycle. (F) Three different dimensionality reduction techniques illustrate the distributional properties associated with the cell cycle.

**Figure 12.**
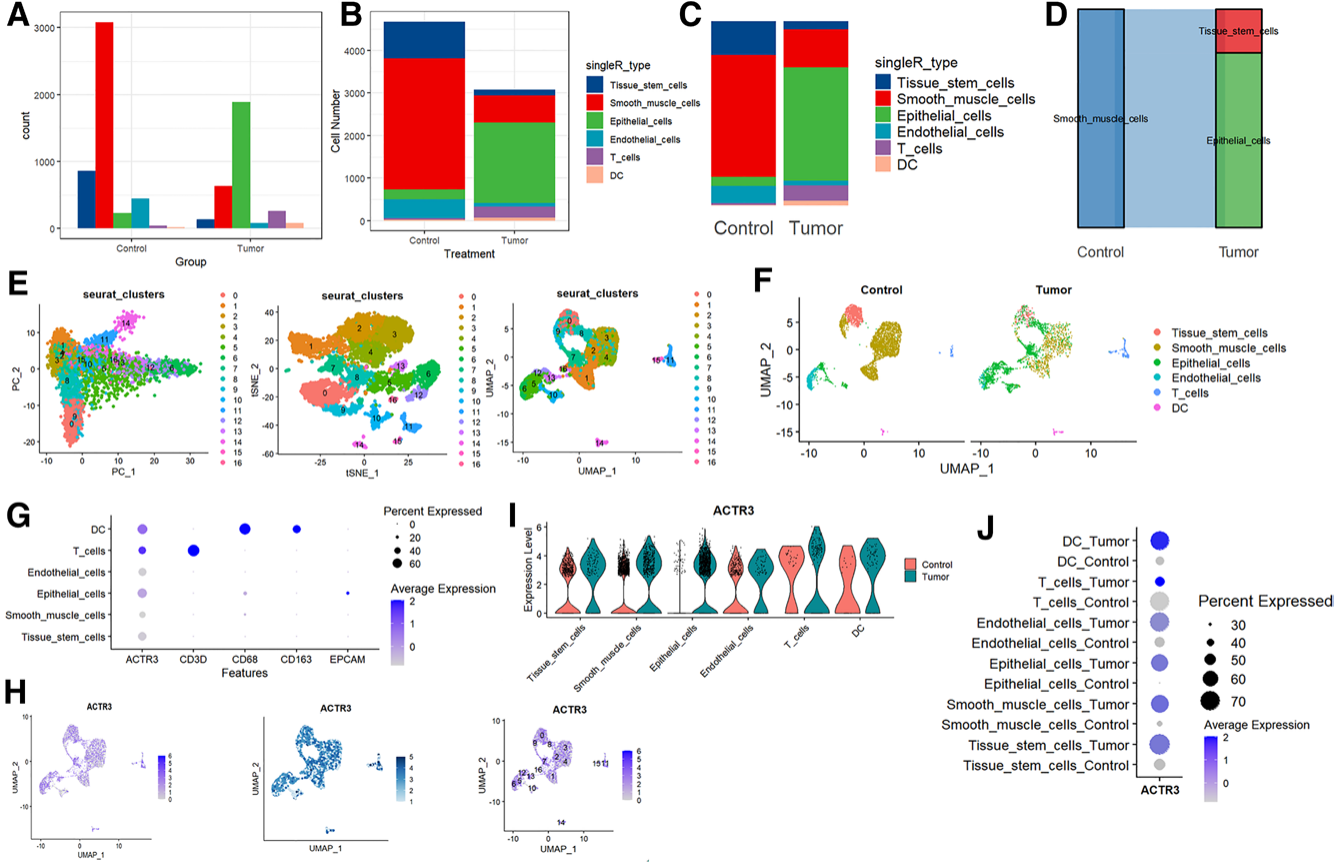
Analysis of single-cell RNA sequencing data from CESC tissues. (A and B) The bar chart, together with the stacked bar chart, depicts the cellular quantities observed in both the control group and the tumor group. (C and D) The allocation of various cell types among the control and tumor groups. (E) The PCA, t-SNE, and UMAP techniques were employed to visualize the 18 unique cellular clusters. (F, G) The clusters received further annotation through the use of designated marker genes. (H) The UMAP visualization illustrates the expression levels of ACTR3 distributed among various cell clusters. (I) Violin plots illustrating the expression levels of ACTR3 across all recognized cell types. (J) The expression of ACTR3 across all recognized cell types. CESC = cervical squamous cell carcinoma and endocervical adenocarcinoma.

The increased expression levels of mitochondrial genes may serve as indicators of potential cellular damage or may initiate apoptosis. Consequently, it is essential to address the influence of mitochondrial gene expression throughout the analysis of single-cell data. For the purpose of cluster analysis, we implemented specific criteria: nFeature_RNA > 200, nFeature_RNA < 2500, and percent.mt < 10, with the intention of minimizing their possible disruption to subsequent analyses (Fig. 11A–C). The Seurat package was utilized to compile marker genes that correspond to the G1, G2M, and S phases of the cell cycle, assigning scores to individual cells to evaluate their phase-specific scores (Fig. 11D). When compared to PCA and t-SNE, which are commonly used for dimensionality reduction, UMAP exhibited superior clustering capabilities due to its direct approach to nonlinear dimensionality reduction (Fig. 11E and F). In the GSE168652 dataset, which comprises cervical cancer tissue samples, we noted a substantial reduction in the number of tissue stem cells and smooth muscle cells within the tumor group, contrasted with a pronounced increase in the number of epithelial cells (Fig. 12A–D). The scRNA-seq data, visualized utilizing PC, t-SNE, and UMAP methodologies, effectively categorized the cells into 16 distinct clusters (Fig. 12E). Each cluster was annotated through the SingleR R package to ascertain cell types (Fig. 12F), which included tissue stem cells, smooth muscle cells, epithelial cells, endothelial cells, T cells, and DC cells. In the context of cell annotation, CD3D is indicative of T cells, while CD68 and CD163 are markers for DC cells, and EPCAM is associated with epithelial cells (Fig. 12G). Coupled with the visualization of ACTR3 expression patterns (Fig. 12H), we observed a notable decline in the overall expression levels of ACTR3 in tissue stem cells and smooth muscle cells within the tumor group (Fig. 12I). Conversely, the average expression levels of ACTR3 in tissue stem cells, smooth muscle cells, endothelial cells, epithelial cells, and DC cells exhibited a significant increase (Fig. 12J).

### 3.10. Experimental validation of ACTR3 expression in cervical cancer

The presence of P63 and p40 positivity serves as a robust indicator of squamous cell differentiation in tumor cells, which constitutes critical evidence for the diagnosis of squamous cell carcinoma (SCC). In the context of cervical neoplasms, the pronounced and widespread positivity of p16 should not be interpreted merely as an overexpression of cyclins; rather, it is regarded as a biological marker indicative of the carcinogenic processes instigated by high-risk human papillomaviruses (most notably HPV16 and HPV18). The E7 protein of HPV instigates unregulated cycles within host cells, resulting in an excessive accumulation of p16 protein (Fig. S1A, Supplemental Digital Content, https://links.lww.com/MD/Q827). Furthermore, the analysis of data derived from TCGA reveals that, taking into account the non-normal distribution of the dataset, the Spearman correlation analysis results demonstrate a noteworthy positive correlation between ACTR3 and both MKI67 (*R* = 0.321) and TP63 (*R* = 0.385) (Fig. S1B, Supplemental Digital Content, https://links.lww.com/MD/Q827).

**Figure 13.**
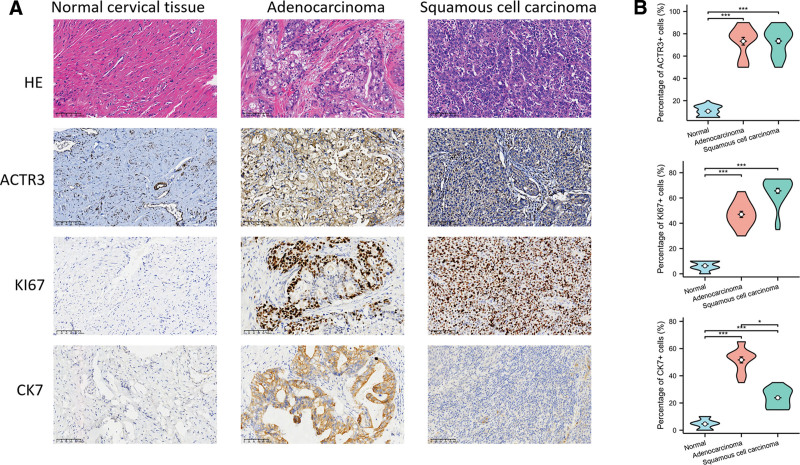
Validation of ACTR3 expression in clinical specimens from patients diagnosed with CESC. (A) The protein expression levels of ACTR3, KI67, and CK7 were found to be significantly higher in CESC compared to those in normal tissue samples. (B) Statistical analysis of ACTR3, KI67, and CK7 positive cells. CESC = cervical squamous cell carcinoma and endocervical adenocarcinoma.

Histological analysis, which employed the well-established technique of HE staining, revealed that the tumor cells associated with cervical adenocarcinoma exhibited histopathological characteristics that were strikingly similar to those found in moderately differentiated gastric-type adenocarcinoma. In contrast, the tumor cells that were identified in cases of cervical squamous cell carcinoma displayed distinct features that are characteristically associated with nonkeratinizing squamous cell carcinoma, highlighting the unique pathological distinctions between these 2 types of cervical malignancies. The protein KI67 serves as a critical biomarker for evaluating tumor malignancy and finds extensive application in both clinical and research environments. An elevation in KI67 expression generally indicates enhanced cell proliferation, elevated division rates, and more aggressive tumor features, all of which correlate with unfavorable prognostic implications. CK7, a protein that is characteristic of intermediate filaments in epithelial cells, is significantly expressed in both adenocytic epithelial tissues and the tumors that originate from them. The findings from the immunohistochemical assessment indicated that, in contrast to normal cervical tissue, there was a marked increase in the expression levels of ACTR3 (*P* < .001), KI67 (*P* < .001), and CK7 (*P* < .001) in cervical adenocarcinoma (Fig. 13A and B). Furthermore, a similar trend was observed in cervical squamous cell carcinoma, where the expression levels of ACTR3 (*P* < .001), KI67 (*P* < .001), and CK7 (*P* < .001) were also significantly elevated when compared to normal cervical tissue.

## 4. Discussion

CESC represents a significant public health challenge, being the third most prevalent cancer among women globally.^[33–35]^ This malignancy not only imposes substantial physiological and psychological burdens on patients but also contributes to considerable mortality rates and economic costs. Despite advancements in vaccination and screening strategies, the limitations of current diagnostic and therapeutic approaches persist, particularly in terms of early detection and treatment efficacy.^[36]^ Consequently, there is an urgent need for innovative biomarkers that can enhance prognostic accuracy and improve patient outcomes. This study aims to address this gap by investigating the role of specific genes associated with the PI3K/Akt/mTOR signaling pathway, thereby providing new insights into the prognostic landscape of CESC. In this study, we focused on the construction of a prognostic risk model for CESC by leveraging bioinformatics analyses and advanced statistical methodologies, including LASSO and Cox regression analyses. Our primary objective was to identify key genes associated with the PI3K/Akt/mTOR signaling pathway that could serve as potential biomarkers for predicting patient outcomes. By integrating gene expression data with clinical outcomes, we successfully identified ten PAMRGs, including ACTR3, ARF1, and PDK1, which demonstrated significant prognostic value. The findings from this research not only highlight the potential of these genes in enhancing prognostic assessments but also pave the way for future investigations into personalized therapeutic strategies for CESC patients.

The findings of our study underscore the critical role of specific genes in the prognosis of CESC, particularly focusing on ACTR3, ARF1, and PDK1. Elevated expression levels of ACTR3 have been consistently associated with poor overall survival across various cancer types, including CESC. This gene is implicated in several biological processes, such as microtubule organization and cilia assembly, which are essential for cellular motility and signaling.^[37]^ The significant upregulation of ACTR3 in CESC tissues compared to normal tissues suggests its potential as a biomarker for tumor aggressiveness and patient prognosis. The correlation of ACTR3 with immune cell infiltration further emphasizes its role in the tumor microenvironment, potentially influencing immune evasion mechanisms that are critical for cancer progression.^[38]^

The PI3K/Akt/mTOR signaling pathway is a critical regulator of cellular processes, including growth, proliferation, and survival, and its dysregulation is implicated in various malignancies, including CESC.^[4,5]^ Our study identified significant associations between the expression of key genes within this pathway and patient prognosis, particularly highlighting the role of ACTR3, ARF1, and PDK1. The upregulation of these genes correlates with poor survival outcomes, suggesting that they may serve as valuable prognostic biomarkers. This finding aligns with previous studies that have demonstrated the oncogenic potential of the PI3K/Akt/mTOR pathway in cancer progression, emphasizing the need for targeted therapeutic strategies that can inhibit this pathway to improve patient outcomes.^[39,40]^ Furthermore, our functional enrichment analysis revealed that ACTR3 is involved in several critical biological processes, including cilia organization and microtubule movement, which are essential for maintaining cellular architecture and function. The association of ACTR3 with neuroactive ligand-receptor interactions and calcium signaling pathways underscores its potential role in modulating tumor microenvironment interactions and cellular signaling dynamics. These findings are consistent with literature indicating that aberrant cilia function can contribute to tumorigenesis and metastasis.^[41]^ Thus, targeting ACTR3 and its associated pathways may provide novel therapeutic avenues for CESC treatment.

Moreover, the relationship between ACTR3 expression and immune cell infiltration presents an intriguing aspect of tumor biology. Our results indicate a negative correlation between ACTR3 levels and the infiltration of various immune cell types, including T cells and NK cells. This suggests that high ACTR3 expression may contribute to an immunosuppressive tumor microenvironment, potentially facilitating tumor growth and metastasis. Previous studies have shown that immune evasion is a hallmark of cancer, and the modulation of immune responses by tumor cells can significantly impact patient prognosis.^[42,43]^ Therefore, understanding the interplay between ACTR3 expression and immune cell dynamics could inform the development of immunotherapeutic strategies aimed at enhancing antitumor immunity in CESC patients. In summary, our findings elucidate the critical role of the PI3K/Akt/mTOR signaling pathway and its associated genes in CESC prognosis. The identification of ACTR3 as a potential biomarker not only enhances our understanding of cervical cancer biology but also opens new avenues for targeted therapies and immunotherapeutic interventions. Future studies should focus on validating these findings in larger cohorts and exploring the mechanistic underpinnings of ACTR3’s role in tumor progression and immune modulation.

The survival analysis results from our study underscore the critical role of specific gene expressions in predicting patient outcomes in CESC. Notably, the LASSO Cox regression analysis identified ten PAMRGs linked to the PI3K/Akt/mTOR signaling pathway, with ACTR3, ARF1, and PDK1 emerging as significant indicators of OS. The Kaplan–Meier survival analysis revealed that patients exhibiting low expression levels of these genes experienced a marked survival advantage compared to those with elevated expression levels, particularly highlighting the adverse prognostic implications of high ACTR3 expression (*P* < .01). These findings align with existing literature that emphasizes the prognostic value of gene expression profiles in various malignancies. For instance, previous studies have demonstrated that elevated levels of ACTR3 correlate with poor survival outcomes in multiple cancer types, including head and neck squamous cell carcinoma and liver hepatocellular carcinoma.^[38,44]^ The ability to stratify patients based on gene expression not only enhances prognostic accuracy but also facilitates the development of personalized treatment strategies. Moreover, the integration of these findings into clinical practice could lead to improved patient management. By identifying high-risk patients through the expression levels of ACTR3 and its associated genes, clinicians can tailor therapeutic interventions more effectively, potentially incorporating early interventions for those identified as high-risk. This approach aligns with the growing emphasis on precision medicine, where treatment is customized based on individual genetic profiles. In conclusion, our study provides compelling evidence that the expression levels of ACTR3 and other PAMRGs serve as vital prognostic markers in CESC, offering a pathway for enhanced patient stratification and personalized treatment approaches. Future research should focus on validating these findings across larger, multi-center cohorts and exploring the underlying biological mechanisms that link these gene expressions to tumor progression and patient survival.

UMAP operates under the uniform manifold assumption, which not only facilitates a strong relationship between neighboring data points but also seeks to preserve the distances among non-neighboring points, or at the very least, prevents them from being excessively close. This property enhances the clarity of the structure within clusters produced by UMAP, showcasing its robust capability for local preservation. Functionally, UMAP serves as a critical enhancement to PCA and t-SNE, the latter of which exhibits limitations regarding efficiency and global structure.^[45,46]^ Additionally, our findings further demonstrate that UMAP significantly surpasses both PCA and t-SNE in terms of clustering performance.

The constraints of this investigation are highlighted by our time-dependent ROC curve analysis, which suggests that one should not depend exclusively on the predictive outcomes derived from a single year. When considering medium- to long-term risk assessments, the existing models appear to be more adept at identifying individuals at medium to high risk over the subsequent 3 to 5 years. However, a thorough evaluation necessitates integration with additional clinical indicators. Future studies should aim to broaden the validation sample size to facilitate repeated assessments of the AUC stability across 1 to 5 years in independent cohorts, thus mitigating the risk of false positives arising from overfitting.

The strengths of the research methods employed in this study lie in the comprehensive integration of bioinformatics analyses, statistical modeling, and clinical data evaluation. By utilizing LASSO Cox regression analysis, we effectively identified a set of ten genes linked to the PI3K/Akt/mTOR signaling pathway, which are pivotal in understanding the molecular underpinnings of CESC. The application of univariate Cox regression and Kaplan–Meier survival analysis further validated the prognostic significance of these genes, allowing for a robust assessment of their impact on patient outcomes. Additionally, the use of large-scale datasets from TCGA and the GTEx project enhances the reliability of our findings by minimizing biases associated with smaller sample sizes. This multifaceted approach not only strengthens the validity of our prognostic risk model but also provides a solid foundation for future investigations into personalized treatment strategies for CESC patients.

In summary, this study highlights the critical role of ACTR3 and its associated genes in the prognosis of cervical squamous cell carcinoma, underscoring the potential of ACTR3 as a valuable biomarker. The findings pave the way for future personalized treatment strategies and prognostic assessments, with the hope of improving patient management and therapeutic outcomes in clinical settings.

## Author contributions

**Conceptualization**: Hongdong Wang, Xu Chen.

**Data curation**: Jianwei Li, Xiujuan Shang.

**Methodology**: Hongdong Wang, Jing Zhang, Xu Chen, Xiujuan Shang.

**Validation**: Hongdong Wang, Jing Zhang, Jianwei Li, Bohao Sun, Xu Chen, Xiujuan Shang.

**Visualization**: Hongdong Wang, Jing Zhang, Xiujuan Shang.

**Writing – original draft**: Hongdong Wang, Jing Zhang, Jianwei Li, Bohao Sun, Yichen Wu, Hao Wang, Xu Chen, Xiujuan Shang.

**Writing – review & editing**: Hongdong Wang, Jing Zhang, Xu Chen, Xiujuan Shang.

## Supplementary Material
