# Supplementary material for: Expression of ACTR3 in cervical cancer and impact on immune cell infiltration and prognosis: A comprehensive analysis based on bulk RNA-Seq and single-cell RNA-Seq
Source: Medicine (Baltimore). 2026 May 12;104(49):e46316. doi: 10.1097/MD.0000000000046316 (PMC12688877; doi:10.1097/MD.0000000000046316)
Supplement: Supplementary file 1 [file medi-104-e46316-s001.docx]

**Figure S1 Cervical squamous cell carcinoma identification**

1. The expression of p16, p40, and p63 in cervical squamous cell carcinoma.
2. The scatter plot shows the correlation between ACTR3 and MKI67, as well as the correlation between ACTR3 and TP63.


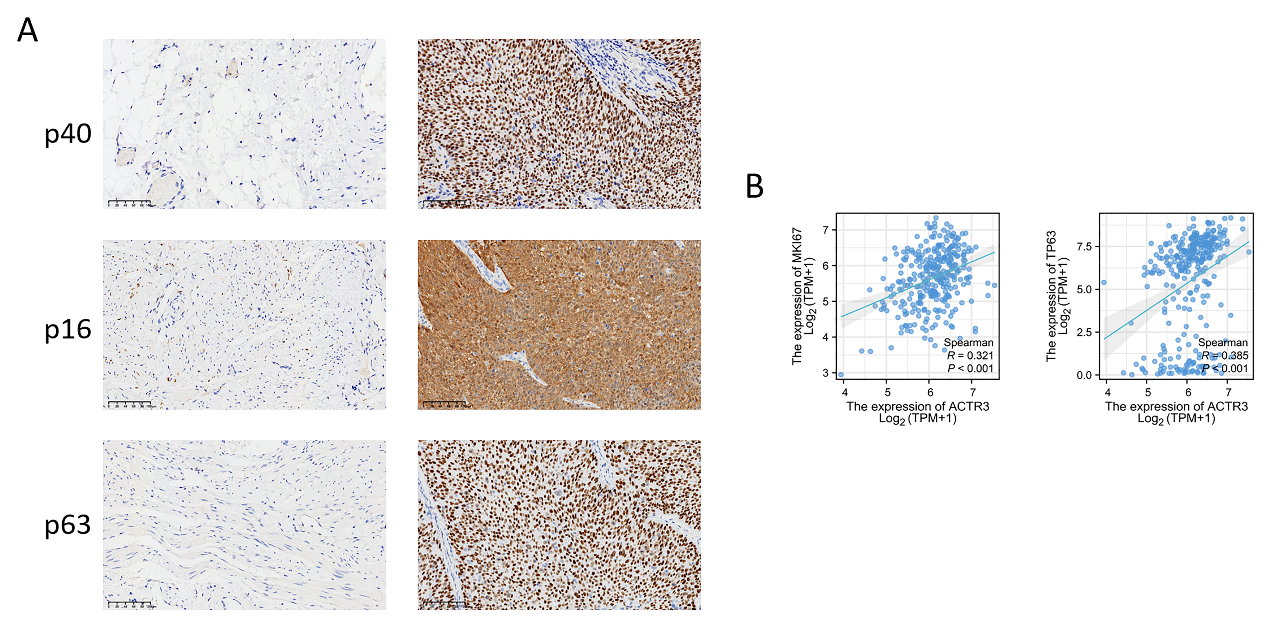


**Table S1 105 PI3K/Akt/mTOR pathway-related genes.**

ACACA

ACTR2

ACTR3

ADCY2

ADRBK1

AKT1

AKT1S1

AP2M1

ARF1

ARHGDIA

ARPC3

ATF1

CAB39

CAB39L

CALR

CAMK4

CDK1

CDK2

CDK4

CDKN1A

CDKN1B

CFL1

CLTC

CSNK2B

CXCR4

DAPP1

DDIT3

DUSP3

E2F1

ECSIT

EGFR

EIF4E

FASLG

FGF17

FGF22

FGF6

GNA14

GNGT1

GRB2

GSK3B

HRAS

HSP90B1

IL2RG

IL4

IRAK4

ITPR2

LCK

MAP2K3

MAP2K6

MAP3K7

MAPK1

MAPK10

MAPK8

MAPK9

MAPKAP1

MKNK1

MKNK2

MYD88

NCK1

NFKBIB

NGF

NOD1

PAK4

PDK1

PFN1

PIK3R3

PIKFYVE

PIN1

PITX2

PLA2G12A

PLCB1

PLCG1

PPP1CA

PPP2R1B

PRKAA2

PRKAG1

PRKAR2A

PRKCB

PTEN

PTPN11

RAC1

RAF1

RALB

RIPK1

RIT1

RPS6KA1

RPS6KA3

RPTOR

SFN

SLA

SLC2A1

SMAD2

SQSTM1

STAT2

TBK1

THEM4

TIAM1

TNFRSF1A

TRAF2

TRIB3

TSC2

UBE2D3

UBE2N

VAV3

YWHAB
